# Rubicon, a Key Molecule for Oxidative Stress-Mediated DNA Damage, in Ovarian Granulosa Cells

**DOI:** 10.3390/antiox14040470

**Published:** 2025-04-15

**Authors:** Kiyotaka Yamada, Masami Ito, Haruka Nunomura, Takashi Nishigori, Atsushi Furuta, Mihoko Yoshida, Akemi Yamaki, Tomoko Nakamura, Akira Iwase, Tomoko Shima, Akitoshi Nakashima

**Affiliations:** 1 Department of Obstetrics and Gynecology, University of Toyama, 2630 Sugitani, Toyama 930-0194, Japan; ky010315@gmail.com (K.Y.); msmito@med.u-toyama.ac.jp (M.I.); katze-n.v.s@hotmail.co.jp (H.N.); t.nishigori.toyama@gmail.com (T.N.); at.furuta523@gmail.com (A.F.); mk.sagamore.hill@gmail.com (M.Y.); au@med.u-toyama.ac.jp (A.Y.); shitoko@med.u-toyama.ac.jp (T.S.); 2 Department of Obstetrics and Gynecology, Nagoya University Graduate School of Medicine, Showa-Ward, Nagoya 466-8550, Japan; tomonakamura@med.nagoya-u.ac.jp; 3 Department of Obstetrics and Gynecology, Gunma University Graduate School of Medicine, Maebashi 371-8511, Japan; akiwase@gunma-u.ac.jp

**Keywords:** autophagy, granulosa cell, infertility, oxidative stress, Rubicon, trehalose

## Abstract

Aging drives excessive ovarian oxidative stress (OS), impairing fertility and affecting granulosa cells (GCs), which are involved in folliculogenesis. This study aims to clarify the relationship between OS and autophagy in GCs and to identify compounds that enhance OS resistance. We identified Rubicon, an autophagy suppressor, as a key mediator of DNA damage in GCs under OS. Hydrogen peroxide (H_2_O_2_) compromised cell viability via DNA damage in the human GC cell line, HGrC1, without affecting autophagic activity. However, autophagy activation increased OS resistance in HGrC1 cells, and vice versa. Among clinically safe materials, trehalose, a disaccharide, protected cells as an autophagy activator against H_2_O_2_-induced cytotoxicity. Trehalose significantly increased autophagic activity, accompanied by reduced Rubicon expression, compared to other carbohydrates. It also reduced the expression of DNA damage-responsive proteins and the production of reactive oxygen species. Rubicon knockdown mitigated OS-induced DNA damage, while Rubicon overexpression enhanced DNA damage and decreased HGrC1 cell viability. Trehalose enhanced OS resistance by activating autophagy and suppressing Rubicon in a bidirectional manner. As Rubicon expression increases in aged human ovaries, trehalose may improve ovarian function in patients with infertility and other OS-related diseases.

## 1. Introduction

Primordial follicles formed until birth enter a state of reduced metabolic activity, halting the development of oocytes and granulosa cell precursors. Once activated, oocyte-secreted factors initiate granulosa cell proliferation, leading to secondary follicle development, in which the oocyte is surrounded by multiple layers of granulosa cells. Secondary follicles are initially less responsive to follicle-stimulating hormone (FSH) but become more sensitive to FSH as they develop more granulosa cell layers. This process occurs when the suppressed expression of the FSH receptor (FSHR) by oocyte-secreted factors is reversed, allowing the outer granulosa cells to express FSHR. As follicles transition from multilayered secondary to early antral follicles, granulosa cells differentiate into mural and cumulus cells. The former converts testosterone to 17β-estradiol, resulting in the expression of luteinizing hormone receptors as preovulatory follicles develop [1]. The latter, which does not express high levels of luteinizing hormone receptors, helps to transport nutrients to the oocyte for enhancing oocyte meiotic progression [2]. Therefore, the proper maintenance of granulosa cell homeostasis is essential for healthy follicular development.

Ovarian aging leads to a decline in both the number and quality of primordial follicles, thereby contributing to reduced pregnancy rates [3]. Clinically, fertility rates begin to decline in women in their late 30s, largely because of poor ovarian response (POR), a condition in which the ovaries exhibit diminished responsiveness to FSH [4]. Oxidative stress increases with age and is the primary cause of ovarian dysfunction [5]. It induces DNA damage, mitochondrial dysfunction, protein oxidation, and lipid peroxidation, all of which contribute to a decline in ovarian function [6]. As oocytes become more vulnerable to external stressors, such as chemotherapy and radiation therapy, their DNA damage repair capacity diminishes [7]. Although a certain amount of oxidative stress is necessary for ovulation, excessive oxidative stress is implicated in infertility-related disorders such as premature ovarian failure, polycystic ovary syndrome (PCOS), and endometriosis. Studies have shown that granulosa cells from women over 38 years of age express lower levels of antioxidant enzymes, such as superoxide dismutase 1 and 2, than those from younger women, resulting in reduced antioxidant capacity [8]. FSH administration stimulates follicular development by targeting granulosa cells expressing FSHR to revive POR ovaries. However, among patients with POR, including aged women, the number of FSHR-expressing follicles is limited, which leads to fewer developed follicles, reduced oocyte retrieval, and lower pregnancy rates. Therefore, alternative treatment methods for FSH administration are required in the clinical setting.

Autophagy is a key cellular mechanism responsible for maintaining homeostasis by producing energy and ensuring the quality control of proteins [9]. Autophagy enables cells to counteract various stressors, such as nutrient deprivation, hypoxia, oxidative stress, and endoplasmic reticulum stress, contributing to enhanced cell survival, anti-inflammatory effects, and proper cellular function [10]. Studies have shown that ovaries lacking Autophagy-related genes exhibit increased follicular atresia, luteal insufficiency, and ovarian atrophy accompanied by p62 accumulation [11,12,13]. Similarly, Atg5-deficient tests show elevated levels of p62 and its phosphorylated form [14]. These findings underscore the importance of autophagy in reproductive function. FSH stimulates the degradation of lipid droplets in porcine granulosa cells through the activation of autophagy via Beclin1 to enhance progesterone production [15], and the conditional knockdown of Beclin1 in granulosa cells impairs mitochondrial progesterone production [16]. Autophagy also facilitates granulosa cell differentiation by degrading WT1 transcription factor, a protein that suppresses FSHR and aromatase expression [17,18]. Additionally, mice deficient in the ectopic P-granule 5 autophagy tethering factor, which is involved in the regulation of autophagy, serve as models for POR, indicating that autophagy is essential for the differentiation of granulosa cells [19]. We also identified bone morphogenetic protein-2 as a factor that promotes follicular development independent of FSH by increasing granulosa cell proliferation in early follicles [20]. Additionally, p62, an autophagy receptor protein, accumulates in the ovarian tissues of patients with PCOS. Exposure to palmitic acid, in combination with the autophagy inhibitor chloroquine, significantly increases reactive oxygen species (ROS) in bovine theca cells [21]. Autophagy failure in ovarian cells may cause ovarian dysfunction.

Autophagic function declines with age, with Rubicon serving as a key autophagy inhibitor that plays a central role in this deterioration [22]. Suppressing Rubicon expression preserves autophagy, mitigates renal fibrosis, and reduces α-synuclein accumulation in the brain, ultimately extending lifespan in animal models. Autophagy has been shown to enhance antioxidant enzyme expression via the p62/NBR1–Keap1–Nrf2 pathway [23,24,25]. However, in renal carcinoma cells, Rubicon inhibits this p62-mediated antioxidant pathway, potentially impairing antioxidant defenses [26]. Regarding infection, Rubicon binds to p22phox, a key component of membrane-bound NADPH oxidase in phagocytes [27], facilitating the phagosomal trafficking of NADPH oxidase, which enhances ROS production, inflammatory cytokine release, and antimicrobial responses in macrophages [28]. In a septic shock mouse model, peptides inhibiting the Rubicon–p22phox interaction significantly reduced ROS levels and cytokine release, lowering mortality [29]. Rubicon also plays a role in ROS regulation in rheumatoid arthritis, a chronic inflammatory disease driven by ROS, where rheumatoid arthritis synoviocytes exhibit increased p22phox and Rubicon expression with colocalization [30]. Under ischemia/reperfusion-induced oxidative stress, Rubicon’s autophagy-inhibitory function is suppressed via its interaction with Caspase recruitment domain-containing protein 9 (CARD9), but in CARD9-deficient conditions, Rubicon-mediated autophagy suppression exacerbates cardiomyocyte injury [31]. Rubicon is further linked to Hippo pathway signaling. Bone morphogenetic protein-2, expressed in the ovary and involved in early folliculogenesis, promotes nuclear translocation of Yes-associated protein 1 (YAP1), the major effector of the Hippo-signaling pathway, suppressing the Hippo pathway and expanding granulosa cell populations [20]. In renal epithelial cells, shear stress activates autophagy via the downregulation of Rubicon expression, which is mediated by nuclear exclusion of YAP1 [32]. However, under pathological shear stress, YAP1 remains in the nucleus, leading to increased Rubicon expression and autophagy inhibition [33]. Thus, beyond its role as an autophagy inhibitor, Rubicon serves as a critical regulator of inflammation, oxidative stress, and cellular homeostasis across various diseases.

In the present study, we identified Rubicon as a regulator of oxidative stress resistance in granulosa cells. Furthermore, the non-reducing disaccharide trehalose, which activates autophagy and downregulates Rubicon expression, has been proposed as a potential strategy for protecting granulosa cells from oxidative stress, thereby contributing to the maintenance of ovarian function.

## 2. Materials and Methods

### 2.1. Cell Culture

A human granulosa cell line, HGrC1 cells, was used as non-luteinized ovarian granulosa cells [18]. The cells were cultured in DMEM (08490-5, Nakalai tesque Inc., Kyoto, Japan) and supplemented with 10% FBS, 100 µg/mL penicillin, 100 µg/mL streptomycin, and 584 µg/mL L-glutamine (56-85-9, Fujifilm Wako, Tokyo, Japan). Cell culture was conducted under conditions of 37 °C, 5% CO_2_, 21% O_2_, and 95% humidity. For drug treatments, cells were seeded at 1.0 × 10^6^ cells/6 cm dish or 4.0 × 10^5^ cells/3.5 cm dish, and reagents were added 24 h after seeding.

### 2.2. Reagents and Antibodies

Tat-Beclin1 D11 (T-B1, NBP2-49888, Novus Biologicals, Centennial, CO, USA) was used as an autophagy activator. Bafilomycin A1 (Baf, 11038, Cayman Chemical, Ann Arbor, MI, USA),chloroquine (CQ, C6628, Sigma–Aldrich, St. Louis, MO, USA), and Wortmannin (Wort, 10010591, Cayman Chemical) were used as autophagy inhibitors. The saccharides were glucose (Glu, 047-31161, Fujifilm Wako), maltose (Mal, 136-00612, Fujifilm Wako), and trehalose (Tre, 206-18455, Fujifilm Wako). Hydrogen peroxide (H_2_O_2_, 081-04215, Fujifilm Wako) was used as an oxidative stress inducer. Primary antibodies used in Western blotting were as follows: Actin (1:10,000, 3700, Cell-Signaling Technology (CST), Danvers, MA, USA), ATG3 (1:2000, TA503346, OriGene, Rockville, MD, USA), ATG5 (1:2000, M153-3MS, MBL Life science, Tokyo, Japan), ATG14 (1:2000, 5504, CST), Ataxia Telangiectasia Mutated (ATM, 1:5000, 2873, CST), p-ATM (Ser1981, 1:5000, 5883, CST), Beclin1 (1:2000, sc48381, Santa Cruz Biotechnology, Santa Cruz, TX, USA), BNIP3L (1:2000, 12986-1-AP, Proteintech, Rosemont, IL, USA), γH2A histone family member X (γH2AX Ser139, 1:2000, 9718, CST), Microtubule Associated Protein 1 Light Chain 3 beta (LC3, 1:2000, 76446, Proteintech), MFN1 (1:4000, 14739, CST), MFN2 (1:4000, 11925, CST), mTOR (1:4000, 2972, CST), p-mTOR (1:4000, 2971, CST), p62 (1:5000, M162-3, MBL Life science), p-p62 (Ser403, 1:2000, GTX128171, GeneTex, Irvine, CA, USA), p95 (1:5000, 14956, CST), p-p95 (Ser343, 1:4000, 3001, CST), Rubicon (1:5000, 8465, CST), Tom20 (1:2000, 42406, CST), and ULK1 (1:2000, 8054, CST), UVRAG (1:2000, M160-3MS, MBL Life science).

### 2.3. Immunofluorescence Staining

This experiment was conducted in the same way as the previous report [34]. Cells were fixed with 4% paraformaldehyde for 10 min, followed by permeabilization with PBS containing 1% Triton X-100 for 20 min. The staining procedure was performed following the manual of the DNA Damage-Detection Kit-γH2AX Green (343-09421, DOJINDO, Kumamoto, Japan). Subsequently, the samples, whose nuclei were stained with Hoechst 33342 (1:10,000, 346-07951, Fujifilm Wako), were observed by fluorescence microscopy (BZ-X800, Keyence, Osaka, Japan) at 20× magnification.

### 2.4. Western Blotting

This experiment was conducted in the same way as the previous report [35]. After washing the cells with cold PBS, cells were lysed and sonicated in RIPA buffer with protease and phosphatase inhibitors. The lysates were centrifuged at 14,000 rpm for 30 min at 4 °C, and protein concentrations were measured using the Bradford method. Blocking was performed with 5% skim milk in Tris-buffered saline with Tween-20 (TBST) at room temperature for 1 h. After washing with TBST, the membrane was incubated overnight at 4 °C with the primary antibody. Following TBST washes, the membrane was incubated with secondary antibodies (anti-mouse IgG HRP-conjugated antibody, 7076, CST, or anti-rabbit IgG HRP-conjugated antibody, 7074, CST) overnight at 4 °C. Visualization was performed using a Multi Imager II Chemi Box (H-674ICE-II, BioTools, Gunma, Japan) and MISVS II software (version 1.0). ImageJ was used for analysis (https://imagej.net/ij/, accessed on 24 April 2024).

### 2.5. Cell Proliferation Assay

This experiment was conducted in the same way as the previous report [23]. To assess the cytotoxicity and cell proliferation, the Premix WST-1 Cell Proliferation Assay System (MK400, Takara, Shiga, Japan) was used following the manual. HGrC1 cells, which were seeded at 8.0 × 10^3^ cells/well in a 96-well plate, were treated with some reagents after 24 h of seeding. After removal of the medium, absorbance was measured after 4 h of WST-1 reagent treatment.

### 2.6. Reactive Oxygen Species (ROS) Assay

HGrC1 cells were seeded at 4.0 × 10^3^ cells/well in a 96-well black chimney plate (655209, Greiner Bio-One, Kremsmünster, Austria) and cultured for 24 h. The procedure was performed following the manual of The ROS Assay Kit-Highly Sensitive DCFH-DA (R252, DOJINDO). After washing the cells with HBSS, fluorescence intensity was measured using a fluorescence plate reader (Spectra Max i3, 5025027A, Molecular Devices, San Jose, CA, USA).

### 2.7. Transfection of siRNA or Plasmids

HGrC1 cells were seeded at 4.0 × 10^5^ cells/3.5 cm dish. After 24 h, Rubicon knockdown siRNA (s18716, s18717, s18718, Thermo Fisher Scientific, Waltham, MA, USA), or control siRNA (4390843, Thermo Fisher Scientific), was introduced by Lipofectamine RNAiMAX (13778030, Thermo Fisher Scientific) at 10 nM concentrations. As for the Rubicon overexpression, pEGFP-Rubicon plasmid (21636, Addgene, Watertown, MA, USA), or pEGFP, as a control was introduced into HGrC1 cells by Lipofectamine 3000 Reagent (L3000015, Thermo Fisher Scientific). After 48 h of treatment, cells were harvested for the experiments.

### 2.8. Collection of Ovarian Tissue

This study was approved by the Ethics Review Committee of the University of Toyama (R2020134) for research using patient specimens. Written informed consent was obtained from patients scheduled to undergo bilateral oophorectomy as part of standard treatment for endometrial or cervical cancer. After oophorectomy, research specimens were collected in a manner that did not interfere with pathological examination. The samples were centrifuged, washed with PBS to remove blood components, and assigned identification numbers. Finally, they were recorded and stored at −80 °C.

### 2.9. Statistical Analysis

Statistical analyses were performed using GraphPad Prism version 10 (GraphPad Software, San Diego, CA, USA). The Mann–Whitney U test was used for comparisons of cell viability. A *p*-value of <0.05 was considered statistically significant (*: *p* < 0.05, **: *p* < 0.01, ****: *p* < 0.0001).

## 3. Results

### 3.1. Autophagy Activity Unaffected by H_2_O_2_ Treatment

**Figure 1:**
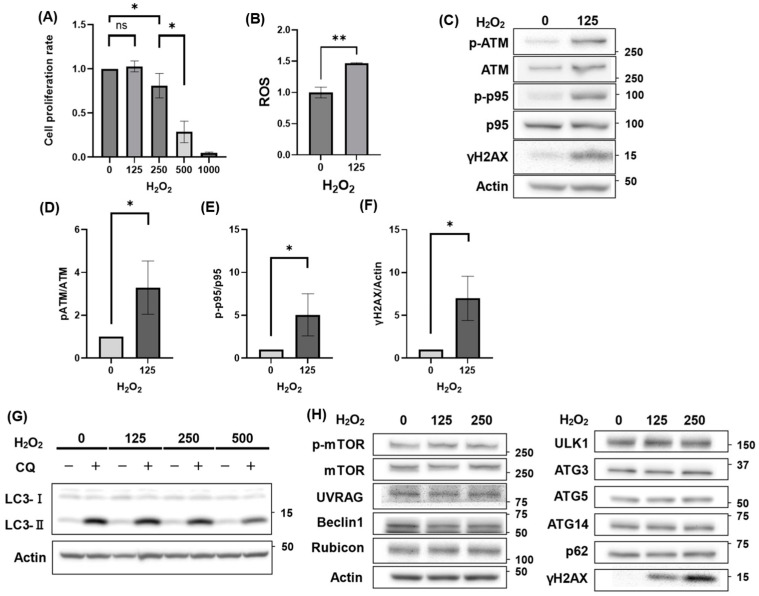
H_2_O_2_ treatment reduced cell viability due to DNA damage but did not affect autophagy in HGrC1 cells. (**A**) Cell viability assessed using the WST-1 assay in HGrC1 cells treated with various concentrations of H_2_O_2_ for 24 h. The Y-axis represented absorbance normalized to the control (set as 1), and the X-axis indicated H_2_O_2_ concentration (µM). (**B**) Reactive oxygen species (ROS) levels of HGrC1 cells treated with H_2_O_2_ treatment at either 0 or 125 µM for 2 h. The Y-axis shows fluorescence intensity normalized to the average value at 0 µM of H_2_O_2_. The X-axis indicates H_2_O_2_ concentration (µM). (**C**) Western blotting (WB) analysis of DNA damage-responsive proteins in HGrC1 cells treated with 0 or 125 µM H_2_O_2_ for 24 h. WBs were shown as follows: p-ATM, ATM, p-p95, p95, γH2AX, and Actin. Actin was used as the control. (**D**–**F**) Quantification of protein expression levels based on the Western blot results shown in (**C**). The graphs represented the relative ratios of p-ATM/ATM (**D**), p-p95/p95 (**E**), and γH2AX/Actin (**F**) under the indicated conditions. All values were normalized to the control group (set as 1). (**G**) Autophagy flux assay in HGrC1 cells. Cells were treated with H_2_O_2_ (µM) for 24 h, followed by chloroquine (CQ, 100 µM) for 2 h before the harvest. (**H**) WB analysis of autophagy-related proteins in HGrC1 cells treated with 0, 125, or 250 µM H_2_O_2_ for 24 h. Western blots were shown as follows: p-mTOR, mTOR, UVRAG, Beclin1, Rubicon, ULK1, ATG3, ATG5, ATG14, p62, and Actin. Results were obtained from at least three independent experiments. Significant difference tests were also performed. Data are expressed as the mean ± S.E. * *p* < 0.05, ** *p* < 0.01, ns: not statistically significant.

We first analyzed the effects of H_2_O_2_ on HGrC1 cell viability. Cell numbers gradually decreased with increasing H_2_O_2_ concentrations, showing a significant reduction starting at 250 µM, and declined dramatically at 500 µM after 24 h of exposure (Figure 1A). Notably, even at 125 µM H_2_O_2_, a concentration that largely maintained cell viability, intracellular ROS levels were already elevated (Figure 1B). This elevated oxidative stress, in turn, led to DNA damage, as evidenced by increased γH2AX expression, suggesting that genotoxic events may precede the significant inhibition of cell proliferation (Figure 1C). The expression of DNA damage-responsive proteins, such as p-ATM and p-p95, was also induced by H_2_O_2_ treatment (Figure 1C). Additionally, we evaluated the induction of DNA damage by measuring the ratios of p-ATM/ATM, p-p95/p95, and γH2AX/Actin, each of which exhibited a significant increase under H_2_O_2_ treatment, confirming an enhanced DNA damage response (Figure 1D–F). Although the impact of ROS on granulosa cells is often related to mitochondrial dysfunction, mitochondrial and mitophagy-related proteins did not change in HGrC1 cells (Supplementary Figure S1). Subsequently, autophagic activity and autophagy-related proteins were evaluated in HGrC1 cells. Autophagy flux assays, which measured the increase in LC3-II by blocking the autophagic flow with CQ, a lysosomal inhibitor, for a short time [36], showed that H_2_O_2_ did not alter autophagic activity (Figure 1G). Short-time exposure to H_2_O_2_, for 3 h, also did not affect autophagic activity (Supplementary Figure S2). In addition, it did not affect the expression of other autophagy markers such as p-mTOR, mTOR, UVRAG, Beclin1, Rubicon, ULK1, ATG3, ATG5, ATG14, and p62. (Figure 1H). Taken together, H_2_O_2_ decreased cell viability due to DNA damage without altering the autophagic activity in HGrC1 cells.

### 3.2. Autophagic Activation Enhanced HGrC1 Viability Against H_2_O_2_-Induced Oxidative Stress

**Figure 2:**
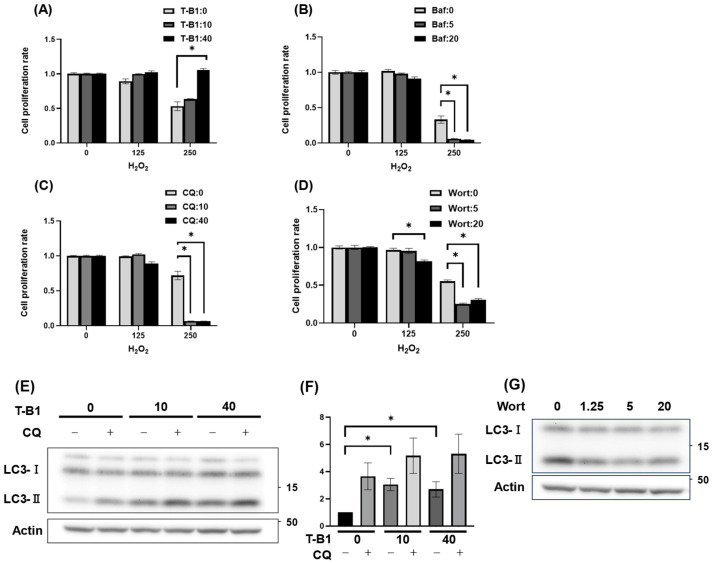
Alternation of autophagic status affected cell viability in HGrC1 treated with H_2_O_2_. (**A**–**D**) Cell viability of HGrC1 cells with H_2_O_2_ (µM) co-treated with Tat-Beclin1 (T-B1) (µM), an autophagy activator, (**A**), or autophagy inhibitors, bafilomycin A1 (Baf, nM) (**B**), chloroquine (CQ, µM) (**C**), or wortmannin (Wort, nM) (**D**) for 24 h. The Y-axis represented absorbance normalized to the control (set as 1). (**E**) Autophagy flux assay in HGrC1 cells treated with T-B1 for 24 h. Cells were treated with T-B1 (µM) for 24 h, followed by chloroquine (CQ, 100 µM) for 2 h before the harvest. (**F**) The graphs showed the expression levels of LC3-II, which were normalized to that of Actin in the treated HGrC1 cells in (**E**). Expression levels were evaluated as the median of three independent experiments. Statistical significance was assessed using the Mann–Whitney U test. (**G**) Western blotting analysis of LC3-II in HGrC1 cells treated with wortmannin (nM) for 24 h. Actin was used as an internal control. Results were obtained from at least three independent experiments. Significant difference tests were also performed. Data are expressed as the mean ± S.E. * *p* < 0.05.

Next, we investigated whether modulation of autophagic activity affects HGrC1 cell viability under oxidative stress caused by H_2_O_2_. As shown in Figure 1A, though cell numbers were gradually decreased with the increase of H_2_O_2_ concentration, co-treatment with T-B1, an autophagy inducer, significantly increased the cell viability (Figure 2A). When the cells were co-treated with autophagy inhibitors [37], Baf and CQ, which suppress lysosomal function and inhibit autolysosome formation, or Wort, which blocks autophagosome formation, Baf and CQ had no significant effect on cell viability at 125 µM H_2_O_2_. However, at 250 µM H_2_O_2_, almost no viable cells remained in the presence of Baf or CQ (Figure 2B,C).

Specifically, when combined with 125 µM H_2_O_2_, 20 nM Wort induced a mild inhibition of cell proliferation. Furthermore, at 250 µM H_2_O_2_, Wort exhibited similar inhibitory effects as observed with CQ or Baf (Figure 2D). Subsequently, we confirmed the effects of these reagents on autophagy. T-B1 treatment significantly increased LC3-II expression levels in the absence of CQ, and this increased LC3-II was further elevated in the presence of CQ, confirming autophagic activation in HGrC1 cells (Figure 2E,F). Wort treatment decreased the expression levels of LC3-II, indicating autophagy-suppressing activity (Figure 2G). The inhibitory activity of CQ was shown in Figure 1G. Considering these results together, autophagic activation boosted resistance to oxidative stress, whereas its inhibition heightened vulnerability, ultimately aiding cell survival under H_2_O_2_ exposure.

### 3.3. Trehalose Activated Autophagy and Decreased Rubicon Expression

**Figure 3:**
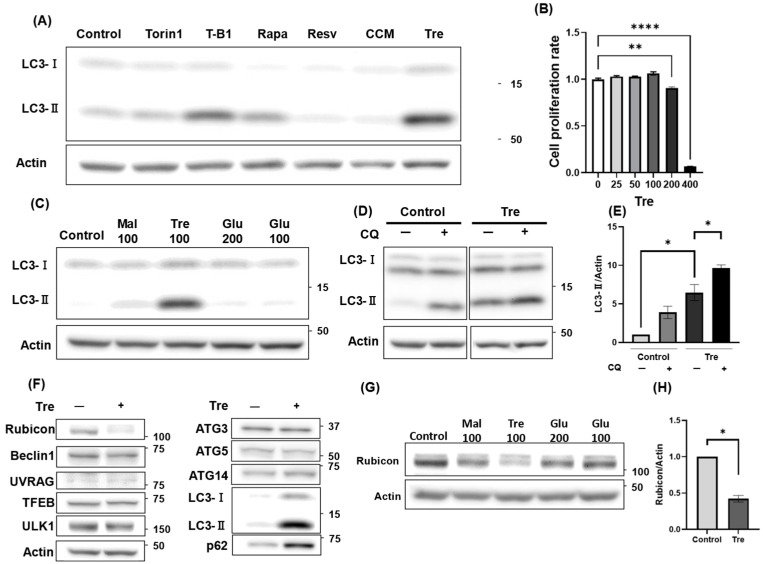
Effects of Trehalose treatment on autophagy-related proteins in HGrC1 cells. (**A**) Western blotting (WB) analysis evaluating LC3 expression levels in response to various autophagy activators in HGrC1 cells. The cells were treated for 24 h with Torin1 (1 nM), T-B1 (40 µM), Rapamycin (Rapa, 1 µM), Resveratrol (Resv, 100 µM), Curcumin (CCM, 10 µM), and Trehalose (Tre, 100 mM). (**B**) Cell viability assessed using the WST-1 assay in HGrC1 cells with various concentrations of Tre for 24 h. The Y-axis represented the mean fluorescence intensity, and the X-axis showed the Tre concentration (mM). (**C**) WB analysis of LC3 expression in HGrC1 cells treated with 100 mM maltose (Mal), 100 mM Tre, or 200/100 mM glucose (Glu) (mM) for 24 h. Actin was used as the control. The control cells were cultured in a medium only. (**D**) WB analysis of autophagy flux in HGrC1 cells treated with Trehalose (Tre) for 24 h. Cells were exposed to 100 mM Tre for 24 h, followed by chloroquine (CQ, 100 µM) treatment for 2 h before harvest. (**E**) Quantification of LC3-II expression based on the WB data shown in (**D**), demonstrating the effect of Tre and CQ on autophagy flux. (**F**) WB analysis of autophagy-related proteins in HGrC1 cells treated with 100 mM Tre for 24 h. WB were shown as follows: Rubicon, Beclin1, LC3, p62, UVRAG, Ulk1, TFEB, ATG3, ATG5, ATG14, and Actin. (**G**) WB analysis of Rubicon expression in HGrC1 cells treated with 100 mM Maltose (Mal), 100 mM Trehalose (Tre), or 200/100 mM Glucose (Glu) for 24 h. Actin was used as the loading control. Control cells were cultured in medium only. (**H**) Quantification of Rubicon expression based on the Western blot data shown in (**G**), comparing its levels across different treatment conditions. Results were obtained from at least three independent experiments. Significant difference tests were also performed. Data are expressed as the mean ± S.E. * *p* < 0.05, ** *p* < 0.01, **** *p* < 0.0001.

To explore safer alternatives to T-B1 for clinical use, we aimed to identify a natural product capable of activating autophagy, thereby minimizing reproductive toxicity (Figure 3A). In this comparative study, HGrC1 cells were treated for 24 h with each product at multiple concentrations that did not reduce cell viability below control levels, and the highest non-cytotoxic dose that maximized LC3-II expression was selected for further analysis. Consequently, trehalose produced the most pronounced increase in LC3-II levels. Although trehalose is commonly used as a preservative and cryoprotectant for oocyte preservation among carbohydrates [38], its cytotoxicity was first evaluated in HGrC1 cells. Trehalose inhibited the growth of HGrC1 cells at 200 mM, and no viable cells were observed at 400 mM trehalose (Figure 3B). Based on these findings, we selected 100 mM trehalose for subsequent experiments, as this concentration was the highest dose that did not compromise cell viability. Trehalose consists of two molecules of α-glucose linked by their hydroxy groups at the first position, whereas maltose consists of two α-glucose molecules linked by their hydroxy groups at the first and fourth positions [39]. Maltose was used as a counterpart to trehalose, as reported previously [40]. Among the carbohydrates tested, trehalose, maltose, and an equal amount of glucose, trehalose significantly increased LC3-II expression levels, while glucose and maltose had little effect on autophagy activation (Figure 3C). The flux assay demonstrated that trehalose treatment elevated LC3-II levels compared to the control, and that these levels increased even further with the addition of CQ (Figure 3D). Correspondingly, the quantitative analysis confirmed a significant increase in LC3-II under trehalose treatment, which was further enhanced following CQ exposure, indicating trehalose-mediated activation of autophagy in HGrC1 cells (Figure 3E). Although mTOR inhibition is known to activate TFEB, its expression levels remained unchanged upon trehalose treatment (Figure 3F). Furthermore, trehalose did not affect the p-mTOR/mTOR ratio in HGrC1 cells (Supplementary Figure S3). Subsequently, we comprehensively examined protein expression in trehalose-treated cells. The expression levels of Rubicon, a known inhibitor of the autophagy pathway, decreased dramatically (Figure 3F). Among the carbohydrates tested, Western blot analysis demonstrated that trehalose induced the greatest reduction in Rubicon expression compared to maltose and glucose (Figure 3G). Quantification further confirmed that trehalose treatment led to a significant reduction of over 50% in Rubicon expression, highlighting its potent suppressive effect (Figure 3H). These results revealed that trehalose activated autophagy and reduced Rubicon expression in HGrC1 cells.

### 3.4. Trehalose Enhanced Cell Viability Against H_2_O_2_-Mediated Oxidative Stress

**Figure 4:**
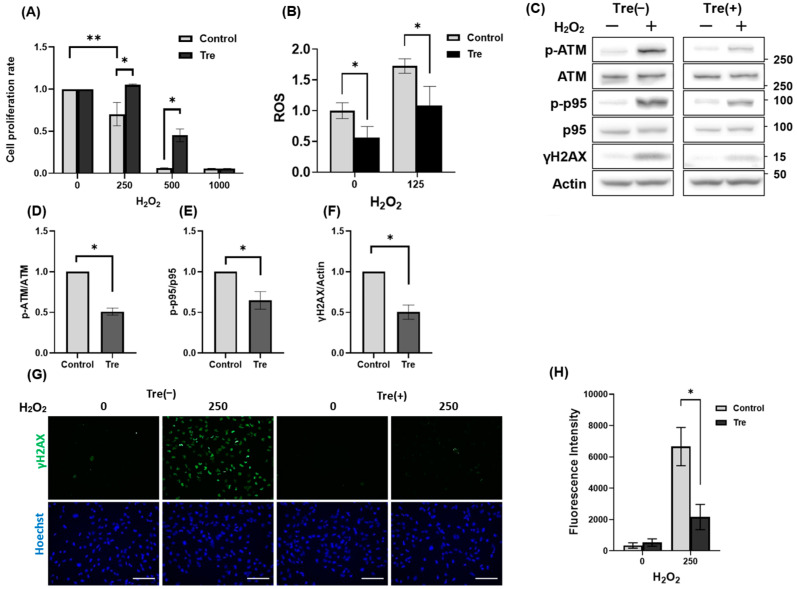
Trehalose-enhanced oxidative stress resistance in HGrC1 cells. (**A**) Cell viability assessed using the WST-1 assay in HGrC1 cells with various concentrations of H_2_O_2_ in the presence of 100 mM trehalose (Tre) for 24 h. The Y-axis represented the mean fluorescence intensity, and the X-axis showed the H_2_O_2_ concentration (µM). (**B**) ROS levels of HGrC1 cells treated with H_2_O_2_ treatment at either 0 or 125 µM for 2 h. The Y-axis shows fluorescence intensity normalized to the average value at 0 µM H_2_O_2_. The X-axis indicates H_2_O_2_ concentration (µM). (**C**) WB analysis of DNA damage-responsive proteins in HGrC1 cells treated with 125 µM H_2_O_2_ in the presence or absence of 100 mM Tre for 24 h. Western blots were shown as follows: p-ATM, ATM, p-p95, p95, γH2AX, and Actin. Actin was used as the control. (**D**–**F**) Quantification of protein expression levels based on the Western blot results shown in (**C**). The graphs represented the relative ratios of p-ATM/ATM (**D**), p-p95/p95 (**E**), and γH2AX/Actin (**F**) under the indicated conditions. All values were normalized to the control group (set as 1). (**G**) Immunofluorescence staining of γH2AX in HGrC1 cells treated with 250 µM H_2_O_2_ in the presence or absence of 100 mM Tre for 24 h. Scale bar, 200 µm. (**H**) The graphs showed the fluorescence intensity of γH2AX based on the images in (**G**). The X-axis showed H_2_O_2_ concentration (µM), and the Y-axis represented the fluorescence intensity. Results were obtained from at least three independent experiments. Significant difference tests were also performed. Data are expressed as the mean ± S.E. * *p* < 0.05, ** *p* < 0.01.

Because T-B1 enhanced HGrC1 cell survival under oxidative stress (Figure 2A), we tested whether trehalose had similar effects. We found that trehalose not only improved cell viability at 250 µM H_2_O_2_ but also significantly ameliorated the more pronounced decrease in viability observed at 500 µM H_2_O_2_ (Figure 4A). Meanwhile, at the relatively lower oxidative stress of 125 µM H_2_O_2_, which maintained comparable cell viability (Figure 1A), ROS accumulation was lower in trehalose-treated cells (Figure 4B). Consistent with these findings, Western blot analysis revealed that trehalose treatment reduced the expression of DNA damage-responsive proteins, including γH2AX, p-ATM, and p-p95 (Figure 4C). Quantitative analysis further confirmed that the p-ATM/ATM, p-p95/p95, and γH2AX/Actin ratios were significantly decreased in the presence of trehalose, indicating its suppressive effect on the DNA damage response (Figure 4D–F). It should be noted that while lower H_2_O_2_ concentrations were sufficient for detecting changes in protein expression via quantitative analyses, a higher concentration, 250 µM, was employed in the immunocytochemistry experiments to achieve a more robust and consistent fluorescence signal for γH2AX, thereby facilitating a clearer assessment of trehalose’s suppressive effects. As a result, immunocytochemistry showed that γH2AX-positive nuclei were reduced by trehalose treatment (Figure 4G). This intensity was also significantly suppressed by trehalose treatment (Figure 4H). Taken together, these results suggest that trehalose mitigated H_2_O_2_-induced oxidative stress by reducing DNA damage, resulting in improved cell viability.

### 3.5. Decreased Rubicon Expression, but Not Autophagic Activation, Was Involved in Reducing DNA Damage

**Figure 5:**
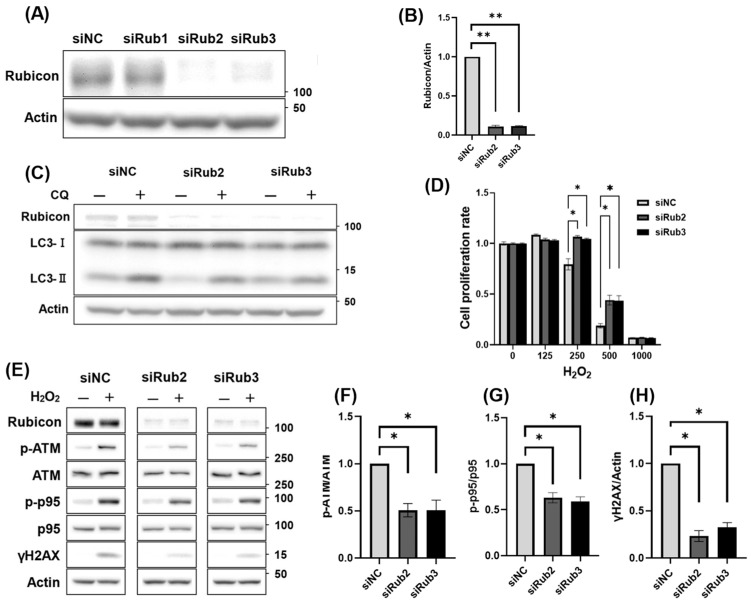
Effects of Rubicon knockdown on autophagy activity and oxidative stress resistance. (**A**) WB analysis of Rubicon in HGrC1 cells to evaluate the levels of Rubicon knockdown using siRNA. Cells were treated with each siRNA at a final concentration of 10 nM for 48 h. siNC represented the negative control, while siRub1, siRub2, and siRub3 corresponded to siRNAs targeting Rubicon. (**B**) Quantification of protein expression levels based on the Western blot results shown in (**A**). The graphs represented the relative expression of Rubicon under the indicated conditions. All values were normalized to the control group (set as 1). (**C**) Autophagy flux assay in HGrC1 cells, which were introduced with siNC, siRub2, or siRub3. After 48 h of siRNA transduction, cells were treated with 100 µM chloroquine (CQ) for 2 h before the harvest. Actin was used as an internal control. (**D**) Cell viability assessed using the WST-1 assay in HGrC1 cells, which were introduced with siNC, siRub2, or siRub3, with various concentrations of H_2_O_2_. The Y-axis represented the mean fluorescence intensity, and the X-axis showed the H_2_O_2_ concentration (µM). (**E**) WB analysis of DNA damage-responsive proteins in HGrC1 cells, which were introduced with siNC, siRub2, or siRub3, treated with 250 µM H_2_O_2_ for 24 h. Western blots were shown as follows: Rubicon, p-ATM, ATM, p-p95, p95, γH2AX, and Actin. Actin was used as the control. (**F**–**H**) Quantification of protein expression levels based on the Western blot results shown in (**E**). The graphs represented the relative ratios of p-ATM/ATM (**F**), p-p95/p95 (**G**), and γH2AX/Actin (**H**) under the indicated conditions. All values were normalized to the control group (set as 1). Results were obtained from at least three independent experiments. Significant difference tests were also performed. Data are expressed as the mean ± S.E. * *p* < 0.05, ** *p* < 0.01.

It is unclear whether the reduction in trehalose-mediated DNA damage is due to a decrease in Rubicon expression, autophagy activation, or both. In addition to the activation of autophagy by trehalose, we investigated whether Rubicon knockdown affected the activation of autophagy and oxidative stress resistance. Using Rubicon-specific siRNAs, we confirmed that siRub2 and 3 decreased Rubicon expression levels by >90% after 48 h (Figure 5A,B). The inhibitory effects of the siRNAs persisted for 120 h after siRNA induction (Supplementary Figure S4). The decrease in Rubicon expression, which was mediated by siRub2 or 3, did not activate autophagic flux (Figure 5C). Since starvation reduces Rubicon expression by activating autophagy in mouse fibroblasts [41], we also investigated whether activation of autophagy by T-B1 decreased Rubicon expression in this cell line. T-B1 did not alter Rubicon expression (Supplementary Figure S5A), suggesting that trehalose influenced the activation of autophagy independently of Rubicon downregulation in HGrC1 cells. Next, we evaluated the relationship between the decreased Rubicon expression and oxidative stress resistance. Decreasing Rubicon expression levels significantly improved cell viability when treated with 250 µM and 500 µM H_2_O_2_ (Figure 5D). Consistent with this, Western blot analysis showed that the knockdown of Rubicon using siRub2 or siRub3 resulted in a marked reduction in p-ATM, p-p95, and γH2AX expression (Figure 5E). These findings were further supported by the quantitative data, which showed significant decreases in the p-ATM/ATM, p-p95/p95, and γH2AX/Actin ratios upon Rubicon knockdown, indicating that reduced Rubicon expression attenuates the DNA damage response (Figure 5F–H). Finally, as for reducing DNA damage by T-B1 treatment, T-B1 did not change the expression levels of DNA damage-responsive proteins (Supplementary Figure S5B). Taken together, these results suggest that the decrease in Rubicon expression, rather than autophagic activation, was responsible for attenuating DNA damage in HGrC1 cells.

### 3.6. Increased Rubicon Expression Enhanced DNA Damage in Granulosa Cell Line

**Figure 6:**
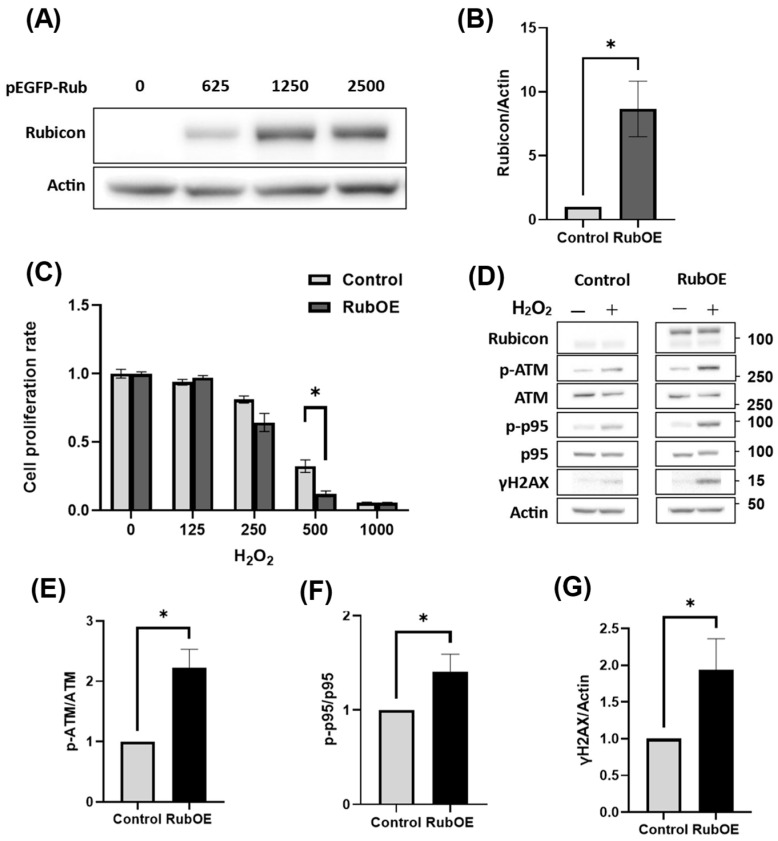
Enhancement of the oxidative stress by Rubicon overexpression. (**A**) HGrC1 cells were transfected with the Rubicon plasmid, and Rubicon expression levels were assessed using pEGFP-Rub. The numbers indicate the amount of plasmid (ng) used for transfection, and Actin served as the loading control. (**B**) For overexpression experiments (OE), 2500 ng of pEGFP-Rub was used, and the Rubicon/Actin ratio was compared with that of the control group. (**C**) Cell viability was evaluated across various H_2_O_2_ concentrations in both the Rubicon-OE and control groups. The Y-axis represented the mean fluorescence intensity, while the X-axis indicates the H_2_O_2_ concentration (µM). (**D**) Western blot analysis was performed to assess DNA damage-responsive proteins in HGrC1 cells transfected with pEGFP-Rub and treated with 250 µM H_2_O_2_ for 24 h. Blots for Rubicon, p-ATM, ATM, p-p95, p95, γH2AX, and Actin (loading control) are shown. (**E**–**G**) Quantitative analysis of the Western blot data in (**D**) was presented as the relative ratios of p-ATM/ATM (**E**), p-p95/p95 (**F**), and γH2AX/Actin (**G**). Values were normalized to the control group (set as 1). Significant difference tests were also performed. Data are expressed as the mean ± S.E. * *p* < 0.05.

To clarify the relationship between Rubicon expression and H_2_O_2_-induced cytotoxicity, we evaluated the impact of Rubicon overexpression on oxidative stress resistance. When H_2_O_2_ treatment was performed for 24 h following transfection with the Rubicon-EGFP plasmid, which induced Rubicon overexpression (Figure 6A,B), cell viability was significantly reduced compared to the control (Figure 6C). Notably, under 250 µM H_2_O_2_, Rubicon overexpression exhibited a trend toward decreased cell proliferation relative to the control, and this inhibitory effect was significantly enhanced when cells were exposed to 500 µM H_2_O_2_. In contrast to the Rubicon-siRNA experiments, Western blot analysis revealed that Rubicon overexpression increased the expression of DNA damage markers, including p-ATM, p-p95, and γH2AX (Figure 6D). Furthermore, quantification of the p-ATM/ATM, p-p95/p95, and γH2AX/Actin ratios demonstrated a significant upregulation of these markers (Figure 6E–G). These results indicated that, unlike Rubicon knockdown, Rubicon overexpression enhanced the inhibitory effect of H_2_O_2_ on cell proliferation by potentiating the DNA damage response.

### 3.7. Increased Rubicon Expression in Ovaries of Postmenopausal Women

**Figure 7:**
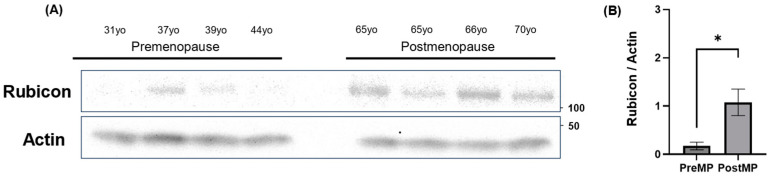
Comparison of Rubicon expression in human ovaries pre- and post-menopause. (**A**) WB analysis of Rubicon in human ovarian tissue. The left four lanes represent premenopausal samples, while the right four lanes correspond to postmenopausal samples. (**B**) The graphs showed the expression levels of Rubicon, which were normalized to that of Actin. PreMP means premenopausal group, and PostMP means postmenopausal group. Significant difference tests were also performed. Data are expressed as the mean ± S.E. * *p* < 0.05.

Rubicon expression increases in some organs with aging. The genetic loss of Rubicon prevents the accumulation of misfolded proteins, which are degraded through the autophagy pathway in neurons, leading to the amelioration of aging phenotypes [22]. Since it is unknown whether Rubicon expression increases in the ovaries with aging, we compared the expression levels of Rubicon in human ovaries between postmenopausal and menstruating women. As expected, Rubicon levels in whole ovarian tissues were significantly higher in postmenopausal women than in menstruating women (Figure 7A,B). Thus, aging affects Rubicon expression in human ovaries.

## 4. Discussion

In the present study, we identified four key findings regarding oxidative stress in granulosa cells. First, both long-term and short-term oxidative stress caused by H_2_O_2_ did not affect autophagic activity. However, autophagic activation enhanced resistance to oxidative stress in the granulosa cell line. Second, trehalose boosts oxidative stress resistance by suppressing Rubicon expression and activating autophagy. Third, Rubicon suppression plays a crucial role in reducing DNA damage, thereby restoring granulosa cell viability against oxidative stress. Finally, Rubicon expression increased in aged human ovaries. However, it is important to note that ovarian function could not be assessed in the young women sampled, representing a limitation of this study. Thus, trehalose contributes to granulosa cell homeostasis by suppressing Rubicon expression in the ovaries. Based on these findings, strategies targeting Rubicon suppression may offer potential treatments for ovarian dysfunction caused by oxidative stress.

Oxidative stress-induced dysfunction in granulosa cells, particularly ROS production, has been well documented. Previous studies have focused on the damage or depletion of mitochondrial DNA [42], showing that lower mitochondrial DNA copy numbers in unfertilized eggs can affect fertility [43]. Additionally, mitochondrial DNA copy numbers in the granulosa cells of patients with PCOS were significantly reduced, likely due to oxidative stress. However, as this study did not show any changes in the mitochondria-related proteins in response to H_2_O_2_ treatment (Supplementary Figure S1), we focused on DNA damage as a cause of cytotoxicity induced by oxidative stress in the granulosa cell line.

Aging exacerbates oxidative stress through DNA damage accumulation [44]. This study showed an increase in Rubicon expression in the ovaries of postmenopausal women. Rubicon levels increase with age in organs such as the kidneys and liver, where they inhibit autophagy [22]. In the gonadal regulation of spermatogenesis, autophagy enhances the degradation of GATA-binding protein 4 (GATA4) to maintain spermatogonial stem cells and germ cell homeostasis in Sertoli cells. The absence of Rubicon in the testis resulted in defective spermatogenesis due to the enhanced GATA4 degradation by autophagy [45]. Applying this finding to ovaries, as GATA4 expression increases with granulosa cell differentiation, increased Rubicon expression, and autophagy inhibition in aging ovaries may lead to aberrant folliculogenesis, which in turn progresses to follicular depletion or may induce excessive oxidative stress following granulosa cell death, leading to ovarian dysfunction. Trehalose may counteract these effects by suppressing Rubicon and enhancing antioxidant defenses. Oxidative stress suppresses Sirtuin 1 (SIRT1) expression, a key regulator of oxidative stress in granulosa cells [46]. SIRT1 suppression also enhances the Beclin1–Rubicon interaction, which inhibits autophagy in tumor cells [47]. Taken together, these results indicate that oxidative stress can inhibit autophagy by enhancing Beclin1–Rubicon binding through SIRT1 suppression in granulosa cells. Applying this hypothesis to our study, we found that oxidative stress did not affect autophagic flux, but trehalose attenuated DNA damage by reducing Rubicon expression without altering Beclin1 levels. Thus, the Beclin1–Rubicon complex may play a central role in the cytotoxicity of oxidative stress in granulosa cells. In other words, disruption of the Beclin1–Rubicon complex attenuated cytotoxicity in the granulosa cell line. The activation of autophagy by T-B1 did not alter the expression of DNA damage-responsive proteins or Rubicon (Supplementary Figure S5A,B) but enhanced oxidative stress resistance. Rubicon contributes to the DNA damage response induced by H_2_O_2_; T-B1-mediated activation of autophagy may enhance oxidative stress resistance through a different mechanism. Therefore, trehalose strongly opposes oxidative stress via dual autophagic regulation.

We focused on DNA damage to explore the mechanisms underlying H_2_O_2_-induced cell death. H_2_O_2_ induces not only single-strand DNA breaks but also double-strand breaks at high concentrations [48]. In cancer cells, the ATM–CHK2–FOXK pathway activates autophagy to mitigate DNA damage, whereas autophagic inhibition exacerbates DNA damage [49]. In the present study, H_2_O_2_ increased double-strand DNA damage, as indicated by ATM phosphorylation, and this damage was exacerbated by autophagy inhibitors such as CQ or Baf. These results suggest that autophagy plays a protective role against DNA damage in granulosa cells. As trehalose or Rubicon knockdown suppressed γH2AX expression, accompanied by reduced ATM phosphorylation, the inhibition of Rubicon mitigated H_2_O_2_-mediated DNA damage. However, the mechanism by which trehalose decreases Rubicon expression remains unclear.

Although it is difficult to fully distinguish these two effects of trehalose, both are likely to work in tandem to maintain granulosa cell homeostasis. Previous studies have shown that Beclin1 knockout in the ovaries results in steroid production failure due to defects in lipid metabolism [16]. Rubicon overexpression in the liver leads to lipid accumulation and organ failure [50]. In humans, Rubicon accumulation, which can occur with aging, may elicit lipid metabolic disorders through autophagy inhibition in the ovaries. Clinically, the senescence markers p16, p21, γH2AX, senescence-associated β-galactosidase, and IL-6, which exhibit the senescence-associated secretory phenotype, were increased in granulosa cells of patients with PCOS [51]. We hypothesize that Rubicon overexpression contributes to ovarian aging. Thus, we are currently evaluating Rubicon expression in aged ovaries or granulosa cells derived from patients with PCOS as a part of future research.

Studies have shown that starvation decreases Rubicon expression levels in mouse cells [52]. However, treatment with autophagy inhibitors such as CQ did not alter Rubicon expression in the current study. Other studies have indicated that DNA damage can increase Rubicon expression levels and that factors contributing to lifespan extension in Drosophila, such as the transcription factor, MondoA, may regulate this process [41]. In Drosophila, the decreased expression of HLH-30, a homolog of TFEB, reduced MondoA expression. Conversely, a decrease in MondoA expression inhibits the nuclear translocation of TFEB, and both are required for autophagy activation [53]. Since trehalose activates TFEB in an mTOR-independent manner [54], it may decrease Rubicon expression via MondoA regulation, a hypothesis that requires further investigation.

Various substances such as melatonin, resveratrol, and metformin have been shown to activate autophagy and reduce oxidative stress [42]. Furthermore, resveratrol and metformin increase SIRT1 expression in granulosa cells [55]. However, resveratrol, which increases SIRT1 expression, also exhibits growth-inhibitory effects on granulosa cells. In contrast, trehalose had the strongest autophagy-activating effect and reduced Rubicon expression without affecting cell viability. Unlike other drugs, trehalose activates autophagy via the Class III PI3K-Beclin1–Rubicon pathway, thereby bypassing the mTOR pathway.

## 5. Conclusions

The expression of Rubicon is increased in aged human ovaries compared to that in women of reproductive age. In this regard, we identified Rubicon as a key regulator of oxidative stress resistance in granulosa cells. Trehalose enhances antioxidant defense by decreasing Rubicon expression and activating autophagy. These findings provide new insights into oxidative stress resistance mechanisms and open the door for the development of ovarian function-preserving treatments using Rubicon-targeted therapies.

## Data Availability

All data generated or analyzed during this study are included in this published article (and its Supplementary Information Files).

## Supplementary Materials

The following supporting information can be downloaded at: https://www.mdpi.com/article/10.3390/antiox14040470/s1, Figure S1: No changes in the mitochondrial proteins in HGrC1 cells treated with H_2_O_2_.; Figure S2: Short-term H_2_O_2_ treatment (3 h) did not induce autophagic activation in HGrC1 cells; Figure S3: Effects of mTOR activation in HGrC1 cells with Trehalose treatment; Figure S4: Sustained knockdown of Rubicon following siRNA treatment in HGrC1 cells; Figure S5: The expressions of Rubicon and DNA damage responsive proteins in cells with Tat-Beclin1.

## Abbreviations

The following abbreviations are used in this manuscript:

ATM	Ataxia Telangiectasia Mutated
Baf	Bafilomycin A1
CARD9	Caspase recruitment domain-containing protein 9
CQ	Chloroquine
FSH	Follicle-stimulating Hormone
FSHR	FSH Receptor
GATA4	GATA-binding protein 4
GCs	Granulosa Cells
γH2AX	γH2A histone family member X
H_2_O_2_	Hydrogen Peroxide
LC3	Microtubule Associated Protein 1 Light Chain 3 beta
OS	Oxidative Stress
PCOS	Polycystic Ovary Syndrome
POR	Poor Ovarian Response
ROS	Reactive Oxygen Species
SIRT1	Sirtuin 1
T-B1	Tat-Beclin1 D11
TFEB	Transcription Factor EB
Wort	Wortmannin
YAP1	Yes-associated protein 1
