# Supplementary material for: Rubicon, a Key Molecule for Oxidative Stress-Mediated DNA Damage, in Ovarian Granulosa Cells
Source: Antioxidants (Basel). 2025 Apr 15;14(4):470. doi: 10.3390/antiox14040470 (PMC12024310; doi:10.3390/antiox14040470)
Supplement: Supplementary file 1 [file antioxidants-14-00470-s001.zip › antioxidants-3491958-supplementary.pdf]

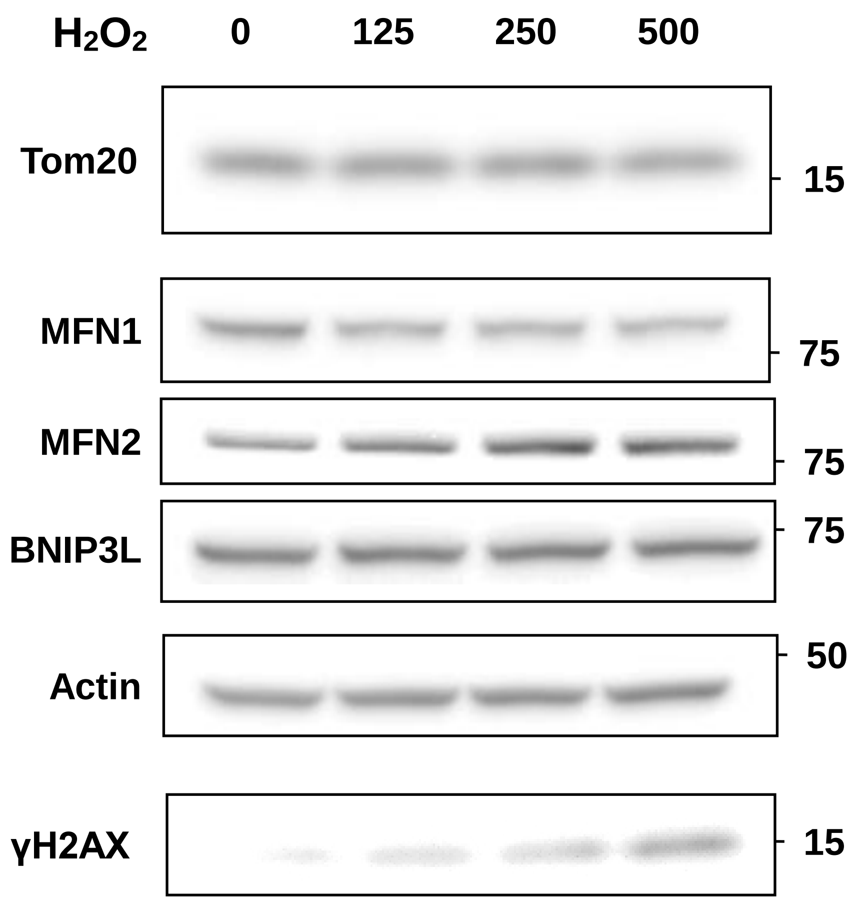

Supplemental Figure S1

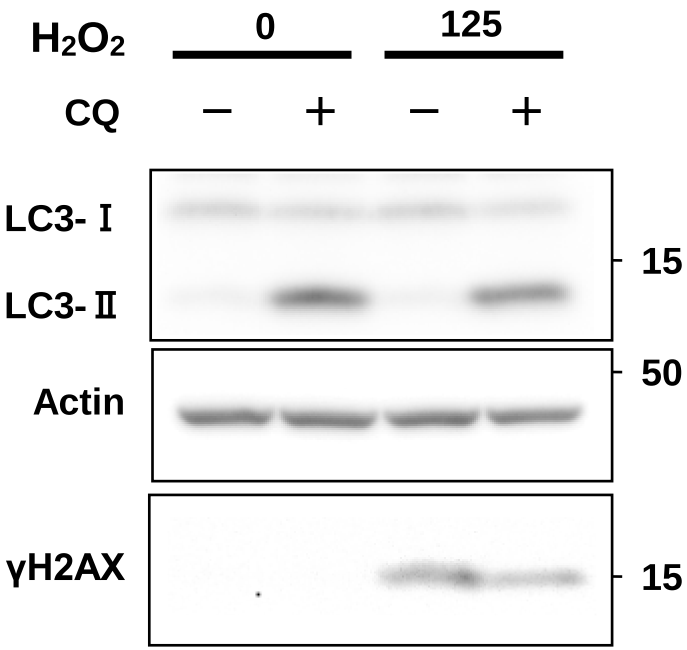

Supplemental Figure S2

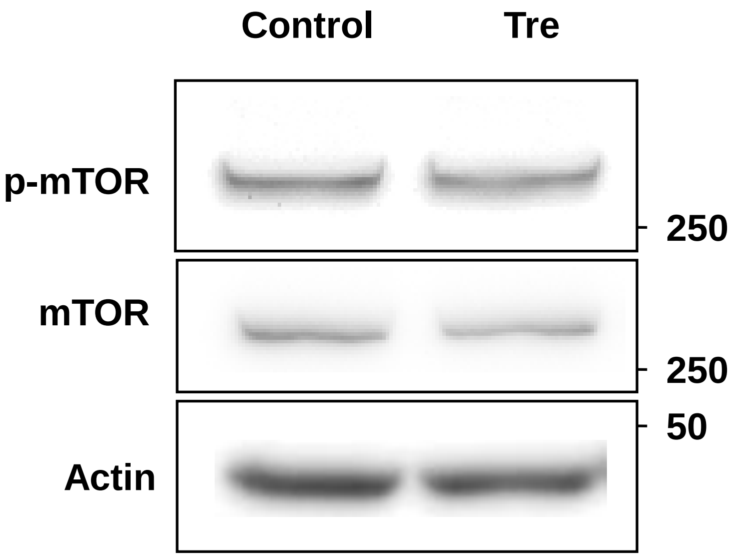

Supplemental Figure S3

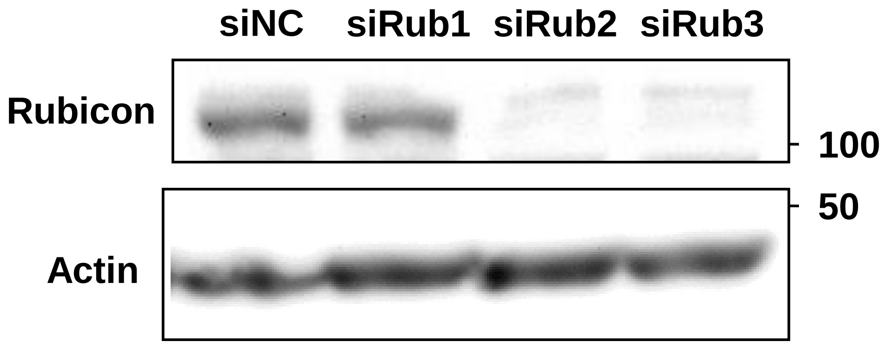

Supplemental Figure S4

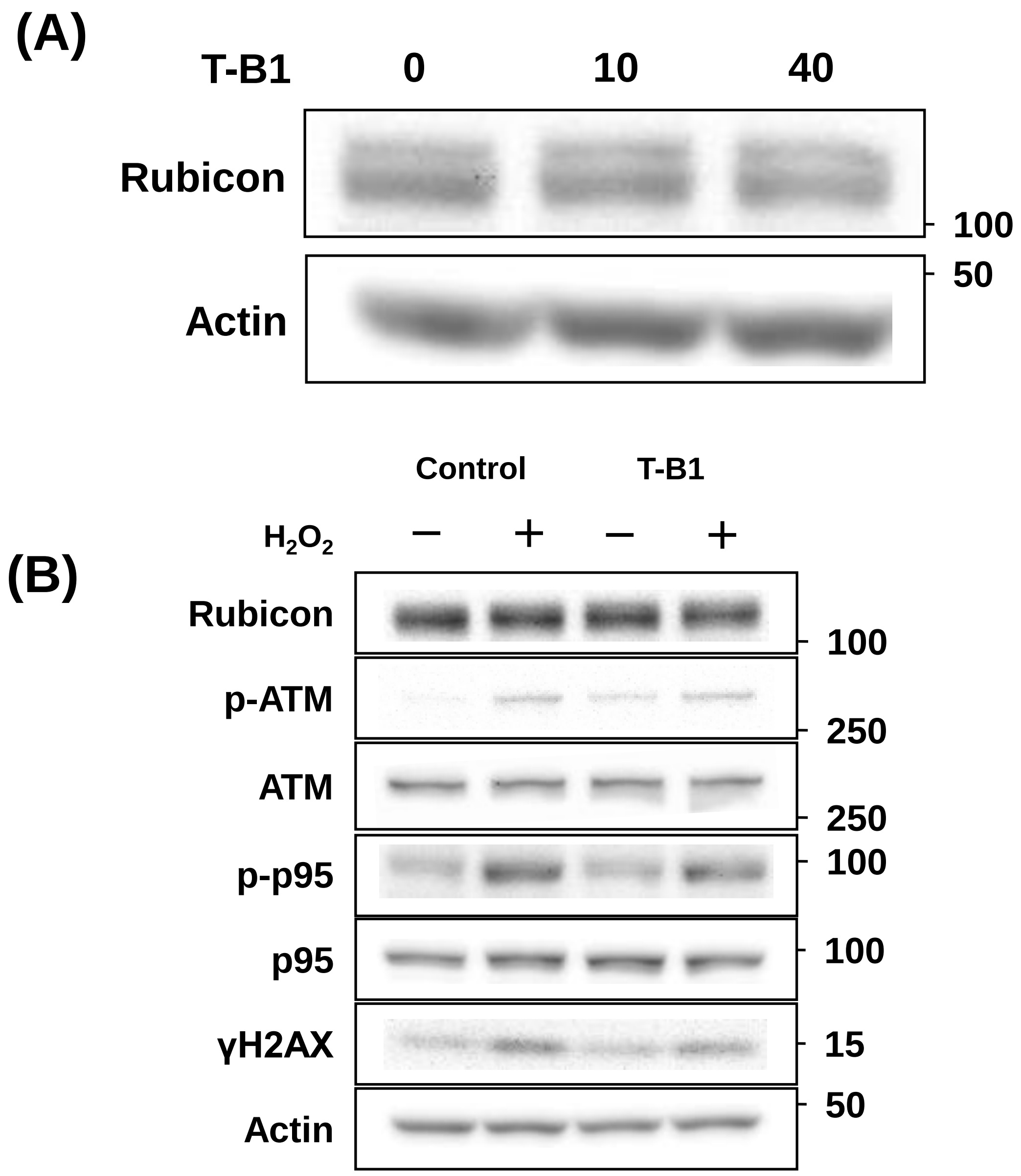

Supplemental Figure S5
